# Protective effects of curcumin and Ginkgo biloba extract combination on a new model of Alzheimer’s disease

**DOI:** 10.1007/s10787-023-01164-6

**Published:** 2023-03-01

**Authors:** Abdel-Azim Assi, Magda M. Y. Farrag, Dalia M. Badary, Essmat A. H. Allam, Mariam A. Nicola

**Affiliations:** 1 Department of Pharmacology, Faculty of Medicine, Assiut University, Assiut, Egypt 71524; 2 Pathology Department, Faculty of Medicine, Assiut University, Assiut, Egypt; 3 Department of Pharmacology and Toxicology, Faculty of Pharmacy, Assiut University, Assiut, 71526 Egypt

**Keywords:** Alzheimer’s disease, Heavy metal mixtures, Scopolamine, Curcumin, Ginkgo biloba extract, Pro-inflammatory cytokines

## Abstract

Alzheimer’s disease (AD) is one of the most prevalent neurodegenerative illnesses, and yet, no workable treatments have been discovered to prevent or reverse AD. Curcumin (CUR), the major polyphenolic compound of turmeric (Curcuma longa) rhizomes, and Ginkgo biloba extract (GBE) are natural substances derived from conventional Chinese herbs that have long been shown to provide therapeutic advantages for AD. The uptake of curcumin into the brain is severely restricted by its low ability to cross the blood–brain barrier (BBB). Meanwhile, GBE has been shown to improve BBB permeability. The present study evaluated the neuroprotective effects and pharmacokinetic profile of curcumin and GBE combination to find out whether GBE can enhance curcumin’s beneficial effects in AD by raising its brain concentration. Results revealed that CUR + GBE achieved significantly higher levels of curcumin in the brain and plasma after 30 min and 1 h of oral administration, compared to curcumin alone, and this was confirmed by reversed phase high-performance liquid chromatography (RP-HPLC). The effect of combined oral treatment, for 28 successive days, on cognitive function and other AD-like alterations was studied in scopolamine-heavy metal mixtures (SCO + HMM) AD model in rats. The combination reversed at least, partially on the learning and memory impairment induced by SCO + HMM. This was associated with a more pronounced inhibitory effect on acetylcholinesterase (AChE), caspase-3, hippocampal amyloid beta (Aβ1-42), and phosphorylated tau protein (p-tau) count, and pro-inflammatory cytokines tumor necrosis factor-alpha (TNF-α) and interleukine-1beta (IL-1β), as compared to the curcumin alone-treated group. Additionally, the combined treatment significantly decreased lipid peroxidation (MDA) and increased levels of reduced glutathione (GSH), when compared with the curcumin alone. These findings support the concept that the combination strategy might be an alternative therapy in the management/prevention of neurological disorders. This study sheds light on a new approach for exploring new phyto-therapies for AD and emphasizes that more research should focus on the synergic effects of herbal drugs in future.

**Graphical abstract:**

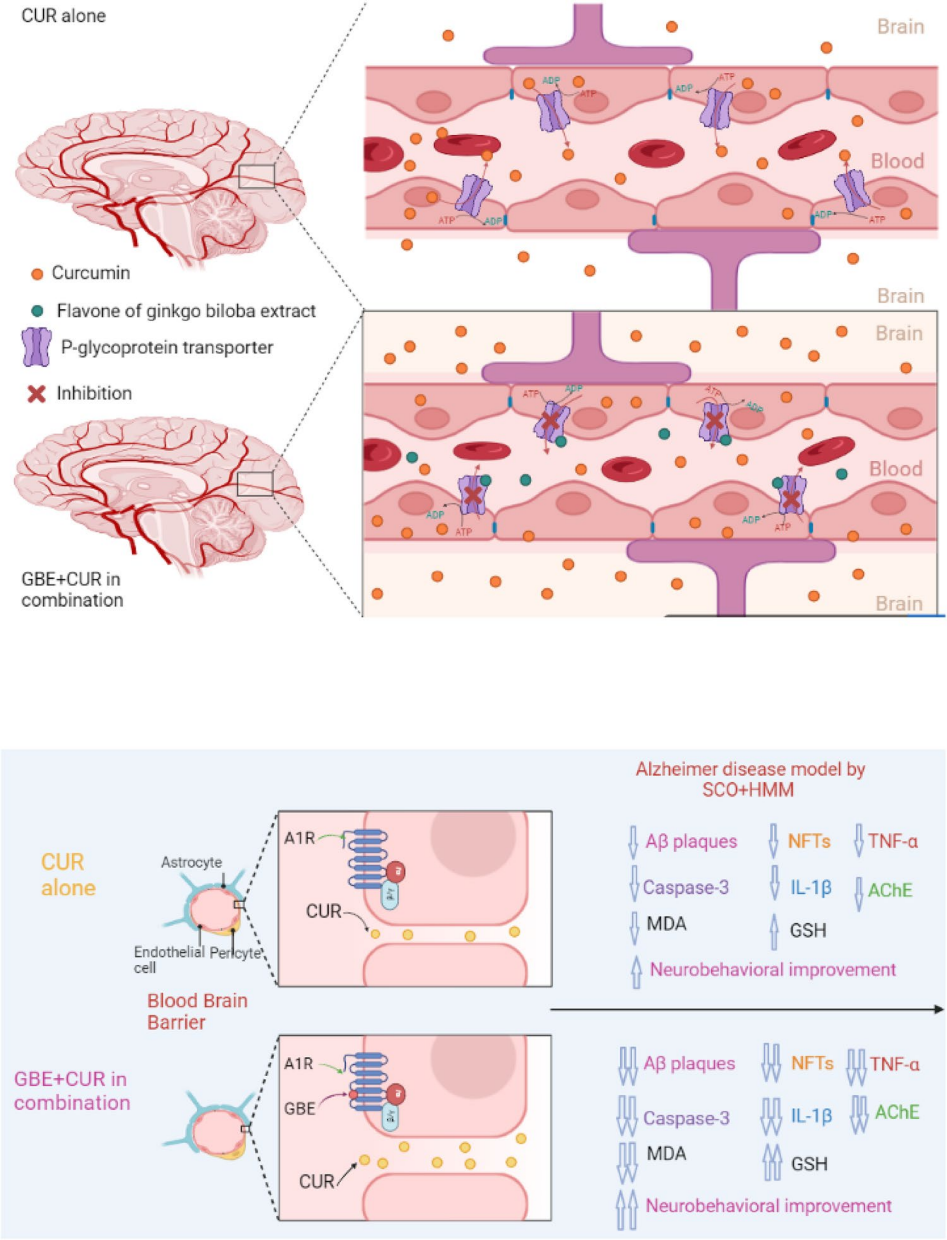

## Introduction

Dementia is a condition in which one’s ability to think, learn, remember, behave, and carry out daily tasks deteriorates (El Gizawy et al. 2021). According to newly released data, there are currently over 50 million individuals living with dementia worldwide, with that number expected to rise to 152 million by 2050 (Li et al. 2022). Dementia has significant social and economic consequences. The current yearly expense of dementia is predicted to be US$ 2 trillion by 2030 (Rodgers 2021). Above all, Alzheimer's disease (AD) is the most common form of dementia, accounting for 60–80% of all dementia cases (Crous-Bou et al. 2017).

Extracellular amyloid beta (Aβ) plaques and intracellular neurofibrillary tangles comprised hyper-phosphorylated Tau protein (pTau) represent the major hallmarks of AD. In addition, the downstream cognitive symptoms can be caused by non-Aβ factors including oxidative stress, inflammation, mitochondrial dysfunction and lipid perturbations (Zhang et al. 2015b).

Despite the increasing incidence and prevalence of the disease, clinical studies on disease-modifying medication have largely failed, making AD therapy one of the most difficult fields of modern medicine (Magierski and Sobow 2015). There are currently six FDA-approved drugs for the treatment of AD, four of which (tacrine, donepezil, rivastigmine, and galantamine) are acetylcholinesterase inhibitors, and one of which (memantine) is a NMDA antagonist, while aducanumab is an amyloid beta-directed monoclonal antibody (Jurcau 2021).

Despite all the scientific advances in recent decades that have increased our understanding of the cellular and molecular bases of AD, still, viable medicines to cure or slow the disease progression are very poor (Cahill and Huang 2017; Abbas et al. 2021).

Considering the dramatic increase in the number of AD cases, as well as the enormous economic and social burden posed on families and societies, advances in AD therapeutic strategies that lead to even minor delays in AD onset or progression would significantly reduce the disease’s global burden (Winblad et al. 2016). This has enticed the researchers in this study to investigate natural replacement therapy, which have fewer side effects and are highly effective in managing AD and memory loss.

Curcumin is the major polyphenol found in turmeric curry (Curcuma longa). Several experimental and clinical investigations have shown that curcumin and its new formulations protect against AD (Antona et al. 2021). Curcumin has been proven to have antioxidant, anti-inflammatory, and neurotrophic properties, as well as the ability to suppress apoptosis and hyper-phosphorylation of tau protein (Yang et al. 2022).

Despite these numerous benefits of curcumin, in research conducted by Begum et al. (2008) and Ringman et al. (2012) on the effects of curcumin supplementation on AD models, it was found that there was no significant impact. The limited capacity of curcumin to cross the BBB, hampers its delivery to the brain at concentrations far less sufficient to offer the expected effects (da Costa et al. 2019). As a result, temporarily opening of the BBB para-cellular route could be useful technique for improving brain medication delivery (Dong 2018).

Ginkgo biloba is a living fossil tree, where its extract (GBE) is currently one of the most researched herbal therapies for cognitive impairments. GBE contains 24% ginkgo-flavone glycosides and 6% terpenoids (Singh et al. 2019; Oken et al. 1998).

According to Liang et al. (2020), GBE potentially activates gap junctions and improves the para-cellular permeability of the BBB via adenosine receptor activation. This could promote the distribution of Ginseng's active components in the brain tissue. Furthermore, previous studies have shown that the flavone in GBE may be a P-glycoprotein inhibitor, which may aid in increasing the concentration of co-administered medicines in the brain (Zhang et al. 2015a; Fan et al. 2009).

Nevertheless, to our knowledge, no reports so far, indicate whether the combination of GBE and curcumin could increase brain concentrations of the later. Consequently, that might improve curcumin’s beneficial effects in ameliorating the cognitive dysfunction and other AD-associated pathologies. Accordingly, the current study focused on the evaluation of the beneficial effects of curcumin and GBE combination on a new modified experimental model of AD, in comparison to conventional standard therapy. Results of this study could provide a novel, effective and safe approach for the prevention and treatment as well as halting the progression of AD.

## Methodology

### Experimental animals

Male Wistar rats (8–10 months old, average weight 300–450g) were purchased and housed in the animal house facility, faculty of Medicine, Assiut University until they were slaughtered. Animals were housed in a 12:12 h light/dark cycle (lights on 7 a.m.) with a regulated room temperature (25 °C) and humidity (65–75%). Rats were housed in groups of six in light plastic cages (60 × 40 × 40 cm). Animals received standard rodent pellet diet and drinking water ad libitum. Animal procedures and their care were carried out under globally established guidelines for the care and use of laboratory animals (Guide for the Care and Use of Laboratory Animals). The tests have been approved by the Research Advisory Ethical Committee of Assiut University's Faculty of Medicine (approval NO. 17101494).

### Drugs and chemicals

Memantine hydrochloride, 98%, was purchased from AK Scientific (USA). Scopolamine hydrobromide trihydrate, 99%, was purchased from ACROS Organics (Belgium). Curcumin, 95%, was purchased from Alfa Aesar (Germany). GBE was purchased from AK Scientific (USA). Arsenic (As), cadmium (Cd), and lead (Pb) heavy metals were purchased from BDH Chemicals Ltd (Poole, England).

Rat TNF-α, IL-1β, AchE and, caspase-3 ELISA kits were purchased from AB-clonal Biotechnology Co., Ltd (USA). Spectrophotometric kits for GSH and malondialdehyde were purchased from Biodiagnostic (Giza, Egypt). Polyclonal Beta-amyloid (Aβ 1–42) antibody and p-tau (Ser202) antibody were purchased from Bioss ANTIBODIES, USA, and Invitrogen Thermo Fisher Scientific, USA, respectively. EconoTek HRP species-specific biotinylated secondary antibody (Goat anti-mouse IgG) was purchased from ScyTek (USA).

### Experimental and treatment schedule

#### Experimental procedure

Cognitive impairment was induced in rats by a newly modified model for AD, using scopolamine hydrobromide trihydrate and a mixture of heavy metals. Scopolamine, dissolved in physiological saline, was intraperitoneally injected (I.P.) at a dose of 4 mg/kg, once a day for 28 days (Assi et al. 2022). Rats received the heavy metals mixture (HMM) through their drinking water throughout the experiment. The HMM was made up of the following components: As 3.80 parts per million, Cd 0.98 parts per million, and Pb 2.22 parts per million (Ashok and Rai 2019; Ashok et al. 2015).

#### Animal groups

Animals were randomly divided into six groups (*n* = 8). Group I negative control (NC), was IP injected with saline and given oral carboxymethyl cellulose (CMC), for 28 days. Group II was injected with IP scopolamine, received oral CMC and HMM-laced water (positive control group; SCO/HMM), for 28 days. Group III received the standard drug; memantine (MEM; 20 mg/kg). Group IV received curcumin (CUR; 100 mg/kg). Group V received a combination of curcumin (in the previously mentioned dose) and ginkgo biloba extract (GBE; 400 mg/kg; CUR + GBE). Oral administration of the vehicle, drug or extracts was given via a gavage, 90 min prior to the IP injection of scopolamine daily for 28 days (the experimental period).

The behavioral tests were conducted on rats from the 21st to the 28th day from the commencement of the experiment, 45 min after the daily scopolamine injection. All animals were sacrificed at day 28 of the study.

### Behavioral tests

#### Passive avoidance task (PA)

The passive avoidance task was tested using equipment consisting of an electric grid floor divided into two equal-sized sections (light and dark) by a sliding door (Ugo Basile, Italy). Rats’ performance is influenced by their inherent preference for darkness. When rats were first placed in the light compartment, the learning trial began. A 2 s duration electric foot shock (1.5 mA) was provided via the grid floor when the rat crossed to the dark compartment. The retention trial was done 24 h following the acquisition trial, with rats placed in the light compartment again, and the latency to enter the dark room (step-through latency; STL) was evaluated using a 300 s cut-off time.

#### Morris water maze (MWM)

The MWM was used to test spatial memory in rats. The animals were permitted to swim freely in a circular pool with a diameter of 1.4 m, filled with opaque water, and conceptually divided into four quadrants. The rats had to find the escape platform, which was submerged 1 cm below the water’s surface and kept in the center of one of the pool’s quadrants. Each rat was given 90 s to look for the hidden platform, after which those who did not find the platform were gently guided and placed on it for 10 s. All animals were subjected to three training trials per day for 6 days. The animals were given a probe trial (retention test) on the seventh day, in which the platform was removed from the tank and the latency to reach the position of the platform and the time spent in the target quadrant were measured.

#### Novel object recognition test (NORT)

The NORT was used to examine non-spatial and long-term memory, which is considered the primary cognitive loss in AD. The test is based on rodents' natural tendency to favor examining novel objects over familiar ones. Rats were kept in the test box for at least 30 min prior to the test. The absence of spontaneous preference was previously evaluated on each duplicate copy of each object. Prior to the test, rats were habituated to the test box without any objects for two days in a row, for a total of 10 min each day (Antunes and Biala 2012). Following a session of habituation, each animal was placed in a test box with two identical objects for as long as it took them to spend a total of 15 s examining these two objects. This was known as the learning trial (familiarization phase). Any rat who did not spend 15 s exploring the objects within the cut-off time (4 min) was not allowed to participate in the study. Exploration is defined as the animal having its head within 2 cm of the object while looking at, sniffing, or touching it (Antunes and Biala 2012). Following the familiarization phase, three testing sessions (test phase) were held to evaluate short-, intermediate-, and long-term memory, at intervals of 5 min, 2, and 24 h, respectively. Animals were exposed to one of the objects previously seen during the familiarization phase along with a novel object, and allowed to explore for 3 min. Animals with low level of object exploring (time spent in exploring novel, plus, familiar objects less than 5 s) were excluded from the data analysis (Antunes and Biala 2012). A discrimination index percent (DI%) was calculated using the following equation: DI% = [(novel object or location exploration time−familiar object or location exploration time)**/**total time spent exploring objects]*100 (Bali et al. 2019).

This result can vary between + 1 and − 1, where a positive score indicates more time spent with the novel object, a negative score indicates more time spent with the familiar object (Antunes and Biala 2012).

#### Preparation of brain homogenate

Animals were sacrificed by decapitation under 2% ether anesthesia on the last day of the behavioral test. Blood samples were collected in tube containing EDTA from the posterior vena cava. Plasma was separated and preserved at − 20 °C until ready for use. Each rat's brain was separated and divided into 2 hemispheres. For immunohistochemical examination, the left hemisphere was fixed in 10% neutral-buffered formalin for 48 h. The hippocampus was dissected on dry ice from the right hemisphere of each rat, and the wet tissues were blotted dry on filter paper, weighed, and stored at − 80 °C for ELISA, HPLC, and spectrophotometric assays.

Upon testing, the hippocampal tissues were homogenized in phosphate-buffered saline (PBS) (pH 7.4) and centrifuged for 10 min to remove debris. Each supernatant was snap-frozen in liquid nitrogen and stored at − 20 °C.

#### Quantification of curcumin in hippocampus and plasma by reversed phase high‑performance liquid chromatography (RP-HPLC)

Curcumin concentrations in the hippocampus and plasma were quantified by reversed phase high-performance liquid chromatography (RP-HPLC). Sample preparation was performed as described previously (Setyaningsih et al. 2016). Briefly, hippocampus tissue was rapidly dissected, weighed, and homogenized in PBS. All the samples were vortexed and then centrifuged for 5 min at 10,000 rpm. The supernatant of each sample was filtered through a 0.22 Μm Millipore filter before the RP-HPLC assay. The RP-HPLC quantifications of curcumin were carried out according to previously described method (Setyaningsih et al. 2016). For each sample, six rats were used. The results are expressed in terms of ng of curcumin per g of tissue.

#### Immunohistochemistry (IHC)

An IHC investigation was carried out to find out how CUR/GBE affected the key elements of senile plaques and neurofibrillary tangles (NFTs) in diseased rats. This technique was used to determine patterns of development of the amyloid beta (Aβ) 1–42 and p-tau (Ser202) proteins in rat hippocampus.

Histological sections (measuring 4.0 mm in thickness) were deparaffinized in xylene, then hydrated in graded alcohols, and the endogenous peroxidase was blocked using 3% hydrogen peroxide in methanol for 5 min. For antigen retrieval, the slides were placed in citrate buffer and put in the microwave for 10 min. For Aβ 1–42 and p-tau (Ser202) staining, sections were incubated with anti-Aβ 1–42 antibody (rabbit polyclonal antibody) (Bioss ANTIBODIES) at a dilution of 1:500 for 2 h at room temperature and anti-p-tau (Ser202) antibody (rat polyclonal antibody) (Invitrogen) at a dilution of 1:2000 overnight at room temperature, respectively. They were then incubated with the corresponding biotinylated secondary antibodies and visualized with chromogen 3–3′-diaminobenzidine and counterstained with hematoxylin stain. Negative control slides were done by omitting the primary antibody. All the assessments were done in a blinded fashion. Photomicrographs were taken by a digital camera (Nikon DMX1200).

The expression of Aβ was discovered as brown patches or plaques in all immuno-stained brain slices, whereas the p-tau protein was identified as a cytoplasmic expression or tiny NFTs. In 10/ HPF (high-power field), the number of Aβ 1–42 plaques or p-tau-labeled NFTs was counted, and the mean number was calculated for each slide and compared among animal groups (Shankar et al. 2009).

### Biochemical assay

#### Analysis of tissue homogenates caspase-3 activity in the hippocampus

An ELISA kit was used to detect the level/activity of caspase-3 as a marker of neurodegeneration in hippocampal homogenate, and the experiment was carried out according to the manufacturer's instructions (Li et al. 2017). Results are expressed in pg/g of tissue.

#### Estimation of acetylcholinesterase (AChE) activity

The hippocampal levels of AChE were measured using ELISA kit (ABclonal Technology), and the absorbance was read at 450 nm using a microtiter plate reader. The concentration of AChE was calculated using standard curves, according to the manufacturer’s protocols (Ellman et al. 1961), and expressed as μg/g of tissue homogenate.

#### Measurement of the hippocampal level of pro-inflammatory cytokines

The goal of this study was to determine the involvement of pro-inflammatory cytokines in the impairment of cognitive function in SCO/HMM rats, as well as the effect of treatment on tumor necrosis factor-α (TNF-α) and interleukin-1β (IL-1β) levels in the hippocampal tissue. The assays were carried out using ELISA kits, as directed by the manufacturer (Barichello et al. 2010).

#### Determination of hippocampal oxidative stress

In addition to reduced glutathione (GSH), the change in the hippocampal level of malondialdehyde (MDA), a lipid peroxidation marker, was employed to assess the effect of treatment on the oxidant/antioxidant balance in the brains of diseased rats as a possible mechanism for improving memory function in these animals.

Glutathione detection was carried out by spectrophotometric kit at 405 nm (Beutler 1963). Results are given in µmol/g tissue. The hippocampal level of MDA was monitored colorimetrically at 534 nm, as described by (Ohkawa et al. 1979). Results are expressed in nmol/g tissue.

#### Determination of curcumin in hippocampus and plasma by reversed phase high‑performance liquid chromatography (RP-HPLC)

Due to curcumin's lipophilic nature, its levels in the hippocampus and plasma are very low and difficult to detect after oral ingestion. Therefore, this study used RP-HPLC which is a sensitive, accurate and reliable method for the determination of curcumin concentration in the hippocampus and plasma.

Chromatographic procedures achieved using Phenomenex, Luna C18 column (150 × 4.6 mm. 5-micrometer particle size) with security guard C18 (4 × 3.0 I’d) and a gradient program of the ideal mobile phase composition.

Internal standard stock solution of quercetin was diluted with methanol to prepare a working stock solution. The frozen samples were thawed at room temperature. After thawed completely, the samples were vortexed for 20 s. To 100 µL of sample, 10 µL of working stock solution of IS was added. Then, 20 µL of 2% acetic acid and 400 µL of acetonitrile were added to induce protein precipitation. The resulting mixture was vortexed for 20 s. A volume of 1000 µL extraction medium, which consisted of 95 vol-% ethyl acetate and 5 vol-% methanol was added to the sample. The mixture was vortexed for 2 min to extract the curcumin. After centrifugation at 1000 rpm for 20 min, the supernatant was removed quantitatively and transferred into 5 mL tubes. The dried samples under vacuum were reconstituted with 100 µL methanol. To remove any undissolved substances, the sample was centrifuged at 8000 rpm for 20 min after which the supernatant was taken. The supernatants were transferred into the inserts of the HPLC amber vials, and an aliquot of 20 µL was injected onto the HPLC column. The method was validated according to the FDA guidelines (Setyaningsih et al. 2016). The method linearity (correlation coefficient of 0.9825) was demonstrated at 1.25–100 ng/ml.

#### Statistical analysis

Data are expressed as the mean ± standard error (SE). Statistical analysis was performed by a one-way analysis of variance (ANOVA), followed by Tukey’s post hoc test, using GraphPad Prism 9.0.0 (GraphPad Software, Inc.). A *P* value < 0.05 was deemed statistically significant for all statistical comparisons.

## Results

### Effect of curcumin and CUR + GBE on learning and memory of SCO/HMM-treated rats

#### Passive avoidance task

**Fig. 1:**
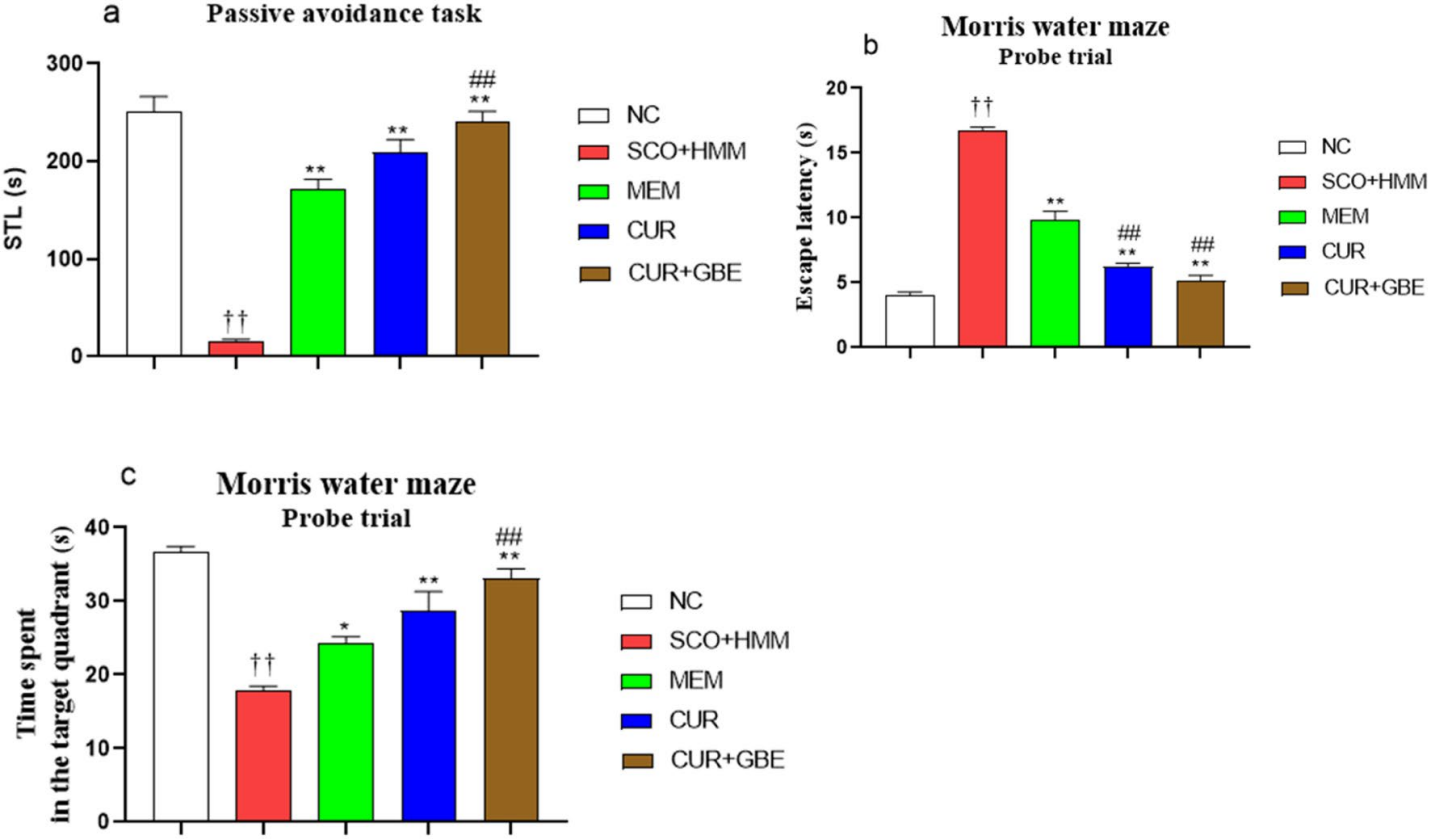
Effect of CUR and CUR + GBE on learning and memory deficits in SCO + HMM rats. **a** Passive avoidance (PA) test. Step-Through Latency (STL) to enter the dark compartment in seconds, 24 h after training, **b** Escape latency to reach previously submerged platform in the Morris water maze (MWM) probe trail following 6 days of the acquisition trail, **c** Time spent on the target quadrant in MWM probe trail where the platform was located. Data are represented as the mean ± SE for *n* = 8. ^††^*P* < 0.01 vs. NC group; *P < 0.05, **P < 0.01 vs. SCO + HMM group; ^##^*P* < 0.01 vs. MEM group. *S* second, *NC* normal control, *CUR* curcumin, *GBE* ginkgo biloba extract, *MEM* memantine, *SCO + HMM* scopolamine/heavy metals mixture

Throughout the acquisition trials, the initial latencies were not significantly different among animal groups. However, in the retention trial, STL in SCO/HMM-treated rats was considerably lower than in the negative control group (250.6 ± 15.82 s for NC vs 16.38 ± 1.438 s for SCO/HMM; *p* < 0.01). Also, rats treated with MEM, CUR, and CUR + GBE had significantly higher STL than rats treated with SCO/HMM (171.3 ± 10.25, 208.8 ± 13.68, 240.0 ± 11.50 s, respectively vs. 16.75 ± 0.2500 for SCO/HMM; *p* < 0.01), (Fig. 1a). Notably, rats treated with the combination had the greatest increase in STL, which was significantly higher than that seen in the groups treated with MEM (*p* < 0.01), while there was no significant difference in STL between rats treated with CUR alone and those treated with the combination.

#### Morris water maze test

During the acquisition phase of this test, a gradual decrease in latency to the escape platform was seen across the 6 days of acquisition, demonstrating that the acquisition was obtained gradually. Rats in the SCO/HMM group showed considerably longer latencies than rats in the other experimental groups (*p* < 0.01). Consistent with these findings, in the probe trial, the latency to determine the position of the formerly submerged platform for the SCO/HMM group was longer than that of the NC group (16.75 ± 0.2500 vs. 4.000 ± 0.2673 s; respectively; *p* < 0.01). Nevertheless, scopolamine-injected, HMM-drinking rats treated with MEM, CUR, and CUR + GBE for 4 weeks, showed a significant reduction in the prolonged escape latency to the position of the platform compared to the SCO/HMM control group (9.875 ± 0.6391, 6.250 ± 0.2500, 5.125 ± 0.4407 s; respectively, *p* < 0.01, vs. 16.75 ± 0.6105 for SCO/HMM (Fig. 1b). Rats treated with CUR + GBE revealed the most pronounced reduction in escape latency to the position of the previously immersed platform, compared to MEM group, while there was no significant difference in escape latency between the group treated with CUR alone and those treated with the combination. Similarly, SCO/HMM rats spent significantly less time on the quadrant where the platform was located than NC rats (17.88 ± 0.6105 vs. 36.63 ± 0.8438 s; respectively *p* < 0.01), whereas this time was significantly longer in rats treated with MEM, CUR and CUR + GBE, compared to SCO/HMM control group (24.25 ± 0.9014, 28.63 ± 2.705 and 33.13 ± 1.288 s; respectively; *p* < 0.01 vs. 17.88 ± 0.6105 for SCO/HMM), (Fig. 1c). The combination-treated rats demonstrated the most prolonged time spent in the target quadrant compared to that of MEM-treated group, while there was no significant difference in the group treated with curcumin only and the group treated with the combination in terms of time spent in the target quadrant.

#### Novel object recognition test

**Fig. 2:**
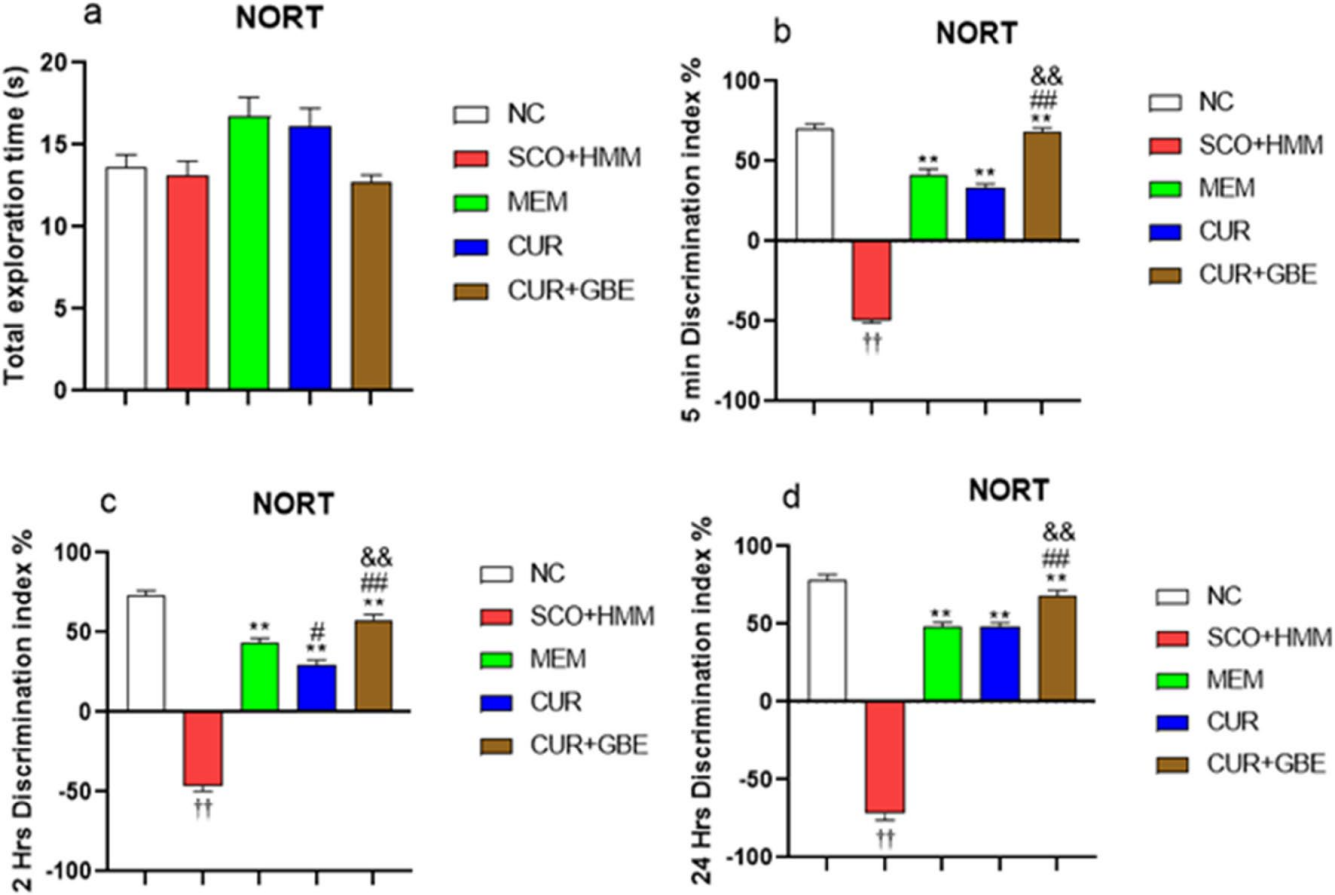
Impact of CUR and CUR + GBE on learning and memory deficits in SCO + HMM rats. **A** Total exploration time for familiar object (in sec) in the NORT. **B**, **C** and **D** % of discrimination index for the novel object 5 min, 2 h and 24 h; respectively, after training in the NORT. Data are shown as the mean ± SE for *n* = 8. ^††^*P* < 0.01 vs. NC group; ^**^P < 0.01 vs. SCO + HMM group; ^##^*P* < 0.01 vs. MEM group; ^&&^*P* < 0.01 vs. CUR group. *S* second, *min* minute, *Hrs*: hours, *NORT* novel object recognition test, *NC* normal control, *CUR* curcumin, *GBE* ginkgo biloba extract, *MEM* memantine, *SCO + HMM* scopolamine/heavy metals mixture

During the training phase, no significant difference was detected in the time spent exploring two identical objects put in diagonal locations among the tested groups, demonstrating that the positioning of the objects had no impact on the exploratory behavior of rats (Fig. 2a). In the testing phase (5 min, 2 h, and 24 h) with two different objects (one novel, the other familiar), diseased rats explore the novel object for a shorter time period and have a lower discrimination index percent (DI%) than normal controls (− 49.04 ± 1.892 vs. 70.09 ± 3.102 at 5 min, − 46.71 ± 3.000 vs. 73.15 ± 3.313 at 2 h, − 71.88 ± 4.112 vs. 78.16 ± 3.621 for 24 h; respectively, *p* < 0.01). Rats treated with natural agents explored the novel item for longer periods of time and had a higher DI% than diseased rats (33.50 ± 2.500 for CUR, 68.24 ± 2.475 for CUR + GBE vs. − 49.04 ± 1.892 at 5 min; 29.81 ± 2.797 for CUR, 57.84 ± 3.234 for CUR + GBE vs. − 46.71 ± 3.000 at 2 h, and 48.35 ± 2.460 for CUR, 68.04 ± 3.867 for CUR + GBE vs. − 71.88 ± 4.112 for 24 h; *p* < 0.01). Similarly, MEM-treated rats explore the novel object for longer periods of time and have a higher DI% than diseased rats (41.69 ± 3.458, 43.10 ± 2.942, 48.30 ± 2.827 vs. at 5 min, 2 h. and 24 h. respectively) (Fig. 2b, c, d). There were substantial differences in the effects of different treatments on DI percent; the group that received a combination of CUR + GBE had significantly higher DI percent than other treated groups; *p* < 0.01. Overall, the findings of the behavioral tests showed that SCO + HMM generated learning and memory deficits in rats, which were recovered using natural agents.

#### Quantification of Curcumin in hippocampus and plasma by RP-HPLC in curcumin alone and CUR + GBE treated rat group

**Fig. 3:**
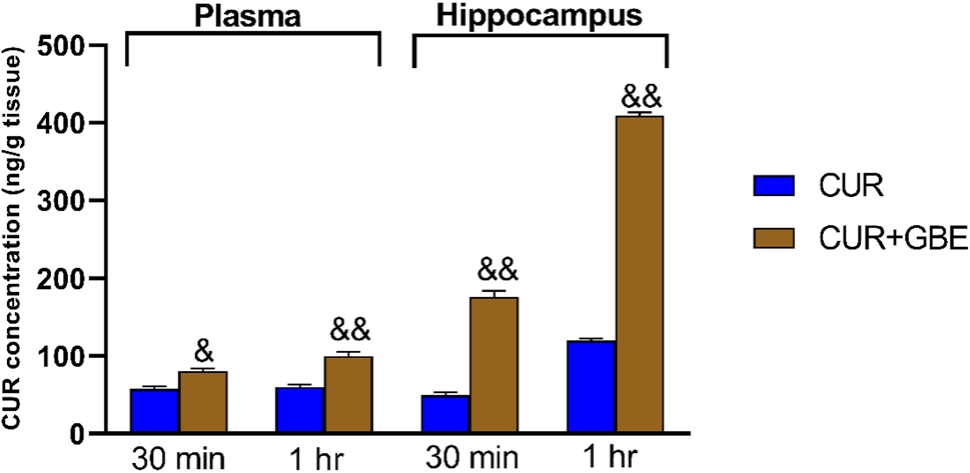
Effect of GBE on curcumin absorption in the plasma and distribution in the brain hippocampus after 30 min and 1 h from oral administration in SCO + HMM Wistar rats. Data are shown as the mean ± SE for *n* = 8. &*P* < 0.05, &&*P* < 0.01 vs. CUR group (unpaired *t* test). *min* minute, *hr* hour, *CUR* curcumin, *GBE* ginkgo biloba extract, *SCO + HMM* scopolamine/heavy metals mixture

The concentrations of curcumin in the hippocampus and plasma were detected by RP-HPLC (Table 1). The data suggest that the combined ginkgo biloba extract and curcumin increased curcumin absorption in the plasma and distribution in the brain hippocampus compared to its administration alone. The curcumin concentration was detected 30 min and 1 h after oral curcumin administration, (Fig. 3) (Table 2).

**Table 1:** Curcumin concentrations in hippocampus (ng/g) and plasma (ng/ml) after single and combined treatment with GBE

	CUR alone 30 min	1 h after CUR alone	CUR + GBE 30 min	CUR + GBE 1 h
Hippocampus (ng/g)	49.46 ± 3.763	119.7 ± 3.069	175.9 ± 8.346	409.5 ± 6.766
Plasma (ng/ml)	57.31 ± 4.134	60.38 ± 2.747	80.58 ± 3.297	100.3 ± 5.463

**Table 2:** Hippocampus/plasma ratio of curcumin concentrations after single and combined treatment with GBE

Hippocampus/plasma ratio (CUR alone 30 min)	Hippocampus/plasma ratio (CUR alone 1 h)	Hippocampus/plasma ratio (combination 30 min)	Hippocampus/plasma ratio (combination 1 h)
0.05349 ± 0.0005978	0.1214 ± 0.006378	0.1571 ± 0.002059	0.2876 ± 0.01726

Data are expressed as the mean ± SE (*n* = 6). CUR: 100 mg/kg curcumin, GBE: 400 mg/kg ginkgo biloba extract

### Biochemical assays

#### Effect of natural agents on AChE activity in the hippocampus of memory deficit rats

**Fig. 4:**
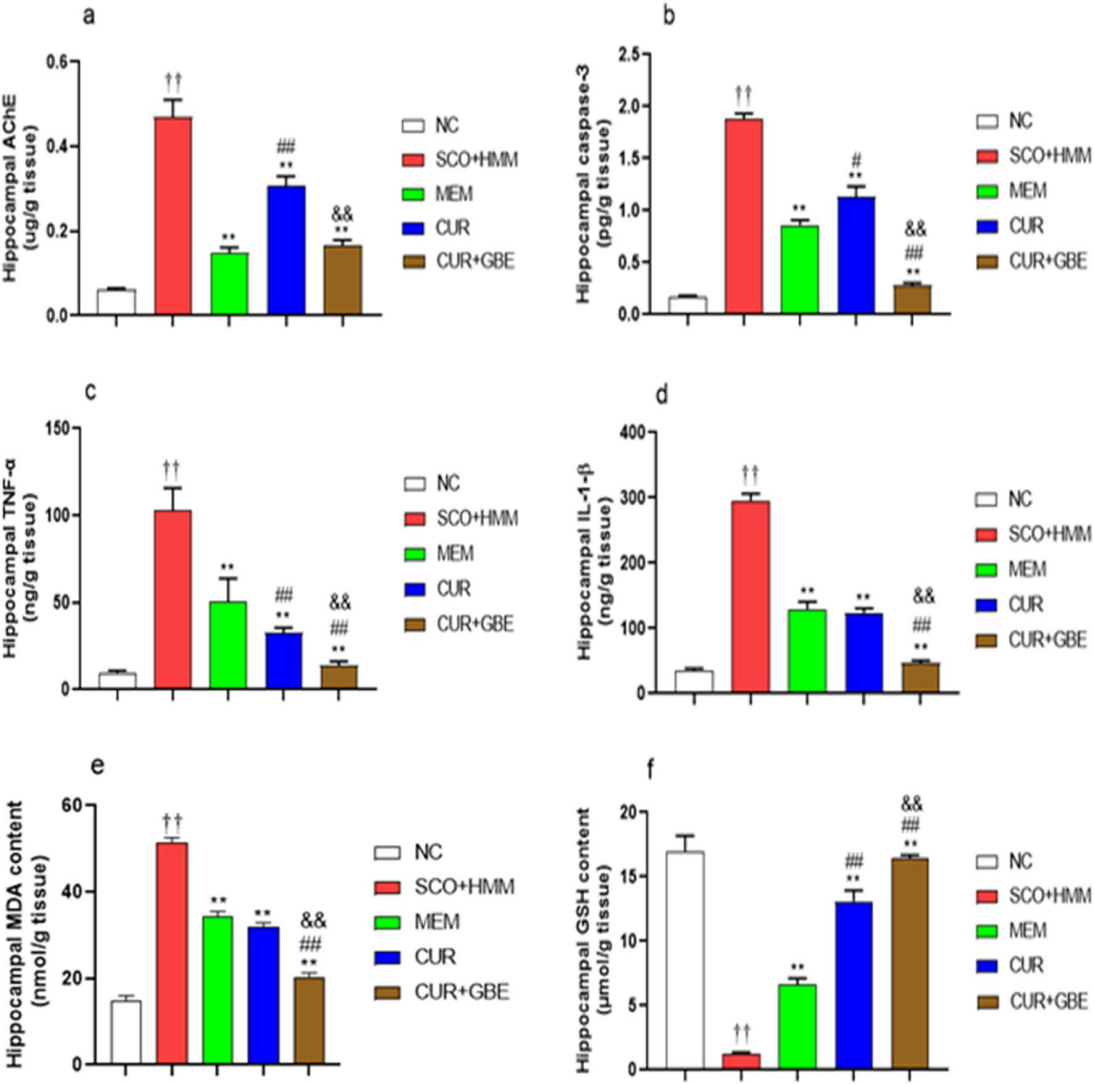
Effect of CUR and CUR + GBE administration on the level of **A** AChE, **B** Caspase-3, **C** TNF-α, **D** IL-1β, **E** MDA, **F** GSH in the hippocampus of SCO + HMM rats. Data are shown as the mean ± SE for *n* = 8. ^††^*P* < 0.01 vs. NC group; **P < 0.01 vs. SCO + HMM group; ^#^*P* < 0.05, ^##^*P* < 0.01 vs. MEM group; ^&&^*P* < 0.01 vs. CUR group. *NC* normal control, *CUR* curcumin, *GBE* ginkgo biloba extract, *MEM* memantine, *SCO + HMM* scopolamine/heavy metals mixture, *AChE* acetylcholinesterase, *TNF-α* tumor necrosis factor-α, IL-1β; interleukine-1β, *MDA* malondialdehyde, *GSH* reduced glutathione

In the hippocampal homogenates of diseased rats that received SCO + HMM, there was a significant increase in AChE activity compared to normal controls (0.4694 ± 0.0410 vs 0.06140 ± 0.0040451 µg/g tissue; respectively, *p* < 0.01). Similarly, as compared to SCO + HMM rats, 28 days of treatment with MEM and natural agents significantly reduced hippocampal AChE activity (0.1475 ± 0.01381 for MEM, 0.3062 ± 0.02340 for CUR, and 0.1652 ± 0.01351 for CUR + GBE, respectively, vs. 0.4694 ± 0.0410 for diseased rats received SCO + HMM; *p* < 0.01), (Fig. 4a). The effects of the CUR and CUR + GBE combination on AChE activity were significantly different.

#### Impact of natural agents’ administration on caspase-3 activity in the hippocampus of diseased rats

The effect of natural substances on neurodegeneration and apoptosis in demented rats was studied by following hippocampal caspase-3 activity. When compared to healthy rats, diseased rats had a significant increase in caspase-3 levels in the hippocampus (0.1677 ± 0.01397 vs. 1.882 ± 0.05015 pg/g tissue; respectively, *p* < 0.01). However, 28 days of treatment to diseased rats with MEM and natural agents reduced the increased level of caspase-3 in comparison to diseased rats that received SCO + HMM (0.8596 ± 0.04733, 1.130 ± 0.1015, and 0.2783 ± 0.02539 pg/g tissue for MEM, CUR, and GBE + CUR; respectively, vs. 1.882 ± 0.05015 ng/g tissue for diseased rats; *p* < 0.01), (Fig. 4b). Strikingly, the combination produced the most noticeable reduction in the hippocampal level of caspase-3 compared to MEM and CUR alone (*p* < 0.01).

#### Effect of administration of natural agents on the hippocampal level of pro-inflammatory cytokines

Diseased rats had considerably higher hippocampal levels of TNF-α and IL-1β than normal control rats (103.1 ± 4.432 vs. 9.741 ± 0.4445 ng/g tissue for TNF-α; respectively, *p* < 0.01; 295.7 ± 10.55 vs.  35.85 ± 3.071ng/g tissue for IL-1β; respectively, *p* < 0.01). In contrast, after 28 days of treatment with MEM and natural agents, there was a significant reduction in the elevated TNF-α levels compared to diseased rats (50.43 ± 4.712, 32.43 ± 1.070, and 13.81 ± 0.8341 ng/g tissue for MEM, CUR, and GBE + CUR; respectively vs. 103.1 ± 4.432 for diseased rats, *p* < 0.01), (Fig. 4c). In the same manner, IL-1β levels notably declined in the hippocampus of animals treated with MEM and natural agents relative to diseased rats (129.6 ± 10.87, 123.6 ± 6.866, and 47.33 ± 3.573 ng/g tissue for MEM, CUR, and GBE + CUR; respectively vs. 295.7 ± 10.55 for diseased rats, *p* < 0.01), (Fig. 4d). Interestingly, the combination produced the most pronounced reduction in the hippocampal level of pro-inflammatory cytokines compared to MEM and CUR alone (*p* < 0.01).

#### Influence of natural agents on the oxidant/antioxidant status in the hippocampus of diseased rats

In the brains of demented rats that received SCO/HMM, the oxidant/antioxidant balance was considerably disrupted, as evidenced by a significant decrease in GSH levels compared to the NC group (1.283 ± 0.1114 vs. 16.98 ± 1.193 µmol/g tissue; respectively, *p* < 0.01) and a marked increase in MDA hippocampal levels (51.32 ± 1.194 vs. 14.78 ± 1.222 nmol/g tissue; respectively, *p* < 0.01). Remarkably, the hippocampal levels of oxidative stress biomarkers were significantly altered in natural agents treated groups in comparison with diseased rats. Levels of MDA in the hippocampus of animals treated with MEM and natural products were significantly lower than that in SCO/HMM rats (34.22 ± 1.304, 32.01 ± 0.8726, and 20.22 ± 0.9931 nmol/g tissue for MEM, CUR, and GBE + CUR, respectively, vs. 51.32 ± 1.194 nmol/g tissue for diseased rats; *p* < 0.01), (Fig. 4e). Also, a marked increase in the reduced GSH levels was observed following 28 days of treatment with MEM and natural agents compared to diseased rats that received SCO/HMM (6.651 ± 0.4639, 13.07 ± 0.8599 and 16.40 ± 0.2961 µmol/g tissue for MEM, CUR, and GBE + CUR, respectively; *p* < 0.01 vs. 1.283 ± 0.1114 µmol/g tissue for diseased rats), (Fig. 4f). Similarly, there were significant differences between MEM, CUR, and GBE + CUR treatments on GSH and MDA, where GBE + CUR showed the most remarkable results (*p* < 0.01).

#### Influence of natural agents treatment on amyloid plaques and tau pathology in SCO + HMM rats

**Fig. 5:**
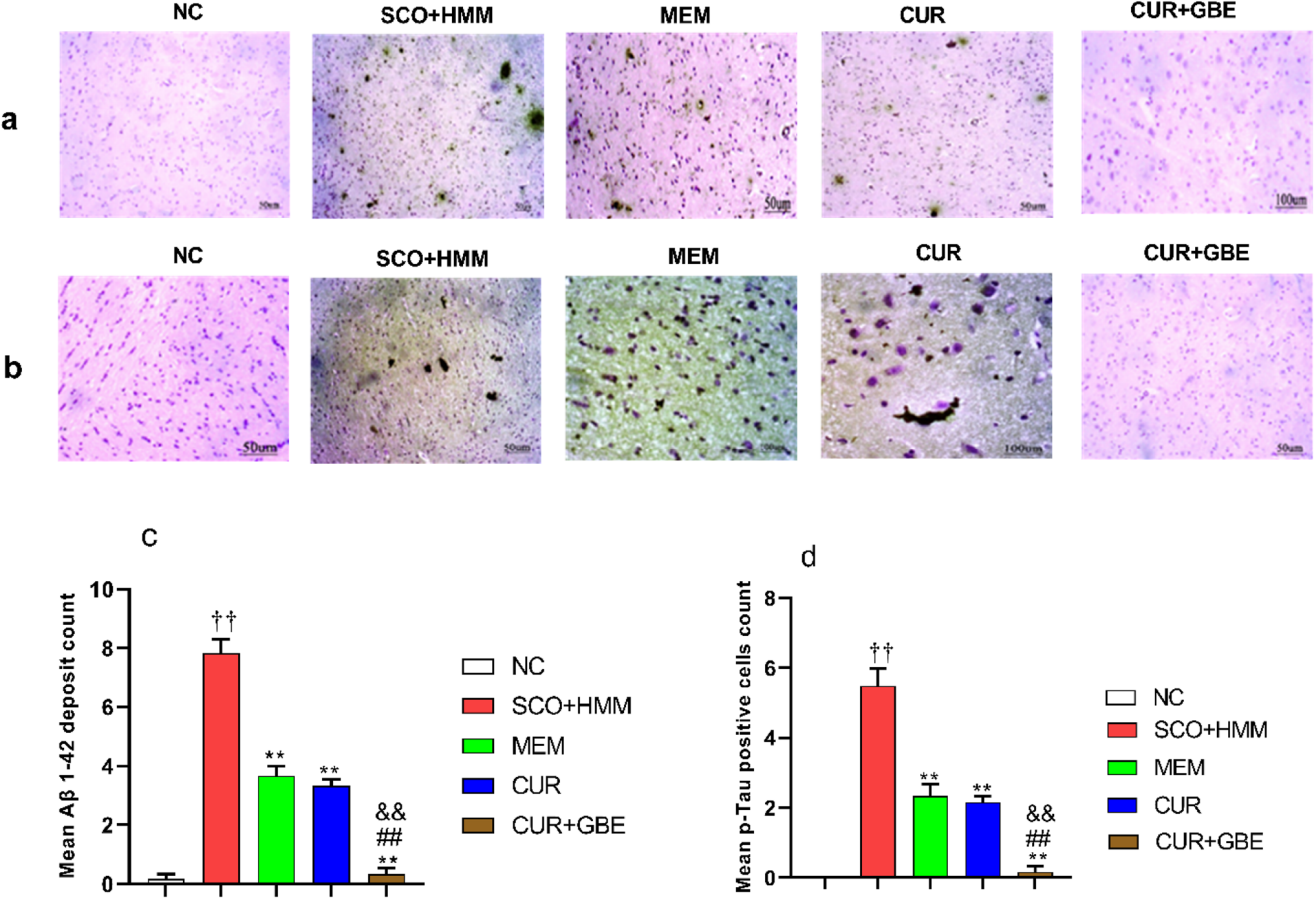
Immunohistochemical examination of AD pathological hallmarks in rat hippocampal tissue. Photomicrographs of Aβ protein deposits **a** and p-tau protein positive cells **b** in brain sections of rats of different groups. Impact of CUR and CUR + GBE administration on count of beta amyloid protein **c** and p-tau **d** in hippocampal tissue in the brain of SCO + HMM rats. Data are shown as the mean ± SE for *n* = 8. ^††^*p* < 0.01 vs. NC group; **p < 0.01 vs. SCO + HMM group; ^##^*p* < 0.01 vs. MEM group; ^&&^*p* < 0.01 vs. CUR group. *NC* normal control, *CUR* curcumin, *GBE* ginkgo biloba extract, *MEM* memantine, *SCO + HMM* scopolamine/heavy metals mixture, *Aβ* amyloid beta, *p-ta*u phosphorylated tau

Immunohistochemical analysis for Aβ and p-tau revealed that the SCO + HMM rats showed a remarkable rise in the mean count of Aβ deposits, together with p-tau (Ser202) positive cells, in the brain, compared to those that received conventional chow (Aβ: 7.833 ± 0.4773 vs. 0.1667 ± 0.1667; p-tau: 5.500 ± 0.500 vs. 0.0 ± 0.0; *p* < 0.01). Compared with the SCO + HMM control group, demented rats that received MEM, CUR, and CUR + GBE for 4 weeks showed a significant decrease in Aβ 1–42 burden in the total brain area analyzed (MEM: 3.667 ± 0.3333, CUR: 3.333 ± 0.2108, and CUR + GBE: 0.3333 ± 0.2108; *p* < 0.01), (Fig. 5a and c). Similarly, p-tau immunoreactivity was considerably lowered by treatment (MEM: 2.333 ± 0.3333, CUR: 2.167 ± 0.1667, and CUR + GBE: 0.1667 ± 0.1667; *p* < 0.01), (Fig. 5b and d). Strikingly, the combination produced the most noticeable reduction in the hippocampal level of Aβ 1–42 and p-tau compared to MEM and CUR (*p* < 0.01).

## Discussion

Dementia is a condition in which one’s ability to think, learn, remember, behave, and carry out daily tasks deteriorates. It is most manifested in AD. Extracellular amyloid beta (Aβ) plaques and intracellular neurofibrillary tangles comprised hyper-phosphorylated Tau protein (p-Tau) represent the major pathological hallmarks of AD. The present study demonstrated the potential role of CUR and GBE combination in attenuating SCO + HMM-induced cognitive deficits in rats. Curcumin was found to affect pathways such as the β-amyloid cascade, tau phosphorylation, neuro-inflammation, and oxidative stress sequelae that are involved in the pathogenesis of neurodegenerative disorders (Witkin and Li 2013; Fan et al. 2017). It was thus concluded that CUR might be used therapeutically to reduce neuro-inflammation in AD. However, curcumin limited absorption as well as permeability through the BBB restricts its therapeutic potential. To achieve acceptable therapeutic results, free CUR can be delivered to target tissues by increasing its bioavailability in the brain and plasma (Anand et al. 2007; Tsai et al. 2011). Thus, our study focused on increasing CUR concentration in the plasma and brain in a trial to enhance its neuroprotective effects on AD parameters.

Based on previous reports on the potential ability of GBE to boost the distribution of the co-administered drugs to brain tissue, the present study investigated the effect of GBE supplementation on CUR plasma and brain concentrations, in addition to its beneficial effects on an AD animal model. This is probably the first study investigating the simultaneous oral administration of CUR with GBE followed by quantifying CUR concentration in plasma and brain using RP-HPLC analysis. The combination achieved significantly higher (*p* < 0.01) levels of CUR in the plasma and brain compared to CUR alone following 30 min and 1 h from the combination oral administration. The enhancement of CUR bioavailability and brain distribution may be attributed to the presence of flavones in GBE. These flavones act as inhibitors of the p-glycoprotein pathway; a significant bottleneck in CUR absorption and distribution. In addition, GBE's capacity to open gap junctions and raise the para-cellular permeability of the BBB via adenosine receptor activation may be another beneficial mechanism. The increased bioavailability induced by GBE is in line with several other studies (Ren et al. 2019; Guo et al. 2020; Mukai et al. 2009). One of these studies found that the concentration of four ginsenosides (the major pharmacologically active components of Ginseng) increased in the brain of rats treated with a combination of Ginseng and GBE (Liang et al. 2020). In contrast, some reports found that GBE decreases the plasma concentration of the co-administered drugs (Hoerr et al. 2022; Xing et al. 2022). The controversy may be due to difference in the type of study, duration of treatment, dose of GBE, co-administered drugs and/or the environment of the study.

Both CUR and GBE have long been known to help treat and prevent AD. Hence, we studied the effect of their concurrent administration on AD-like alterations. Due to its numerous limitations to mimic human AD, SCO model was used in the present study with some modifications. Ashok et al. (2019) found that rats receiving a mixture of As, Cd and Pb daily in drinking water developed remarkable AD-like alterations. Therefore, the present study used a modified SCO model by addition of HMM to enhance the pathological features of the traditional SCO model. Our novel model met the requirements for AD induction, and this was confirmed by significant memory decline, elevation in brain lipid peroxidation biomarkers, Aβ1-42 deposits, p-tau (Ser202) positive cells, TNF-α and IL-1β, as well as caspase-3 and AChE activity. On the other hand, there was a significant decrease in GSH level. Similar findings from other researches conducted on SCO model support these outcomes (San Tang 2019; Ashok et al. 2015).

Many previous studies reported that CUR alone has antioxidant and anti-inflammatory activities, decreases Aβ1-42 deposits, p-tau (Ser202) aggregation, decreases caspase-3 hippocampal levels and acts as anti or decreases cholinesterase activity, with concurrent improvement in the cognitive function in different AD animal models induced by many substances including SCO (Lin et al. 2020; Huang et al. 2016; Akinyemi et al. 2017; Banji et al. 2014).

To examine the impact of CUR alone and CUR and GBE combination, the following tests (the PA, MWM, and the NORT) were chosen to evaluate the behavioral changes in different groups of rats. These tasks are widely used to evaluate learning and memory (Morris, 1984, Rasmussen et al. 2001; Botton et al. 2010). The study found that SCO + HMM-treated rats increased the escape latency of the probe trial of MWM but decreased time spent in the target quadrant, as well as the STL of PA, and time for exploration of the novel object in addition to the DI%.

In contrast to SCO + HMM-treated rats, CUR + GBE-treated animals, significantly lengthened STL in the PA task, demonstrating their facilitation of the cognitive functions in the retrieval stages of memory. In addition, rats treated with these natural products showed a significant reduction in the escape latency, while spending noticeably more time in the target quadrant in the MWM probe trial. Similarly, the treated groups explored the novel object for a longer time with markedly higher DI% than the diseased rats. These findings confirm that these natural agents enhanced learning and recognition memory in the NORT and reversed cognitive function impairment of diseased rats induced by SCO + HMM. Strikingly, the combination-treated rats showed a significant increase in DI% compared to the CUR-alone group. In harmony with our results, Zhao et al. (2021) found that rats that co-administered GBE and low/high-dose donepezil had significantly shorter escape latency and more time was spent in the target quadrant than those treated with individual low/high-dose donepezil or GBE. Moreover, the study of Abdelmeguid et al. (2021) found that aluminum chloride /D-galactose AD rats showed significant increase in the DI % when treated with docosahexaenoic acid plus GBE.

Our results also revealed that SCO + HMM rats that received CUR and GBE combination for 4 weeks showed a significant decrease in Aβ1-42 burden and p-tau immunoreactivity in the total brain area analyzed. Notably, no previous studies reported the effect of GBE, in combination, on AD pathological hallmarks. However, GBE alone has a proven efficacy on theses hallmarks in previous reports (Nikmahzar et al. 2018; Zeng et al. 2018; Verma et al. 2020).

Numerous investigations have revealed that SCO induces brain oxidative stress. Likewise, human AD brains have elevated levels of ROS (reactive oxygen species). Consequently, scavenging oxygen free radicals is one of the most essential criteria for an AD modifying drug (Morrison et al. 2010; Wang et al. 2016). Results of the present study revealed that combination of CUR and GBE resulted in a significant decrease in the hippocampal levels of MDA, with a concomitant increase in GSH, compared to SCO + HMM diseased rats. Interestingly, the combination produced a more pronounced effect on the oxidative stress parameters than CUR alone. In accordance to our results, Zhao et al. (2021) found that rats treated with low/high-dose donepezil and GBE had significantly lower MDA activities than those treated with either low/high-dose donepezil or GBE alone. Additionally, the study of Tian et al. (2013) demonstrated that hyperbaric oxygen/GBE combination showed more pronounced effects in improving GSH activity and reduced MDA content.

Microglial cells in the CNS trigger inflammatory processes by releasing cytokines in response to APP (amyloid precursor protein), which leads to a continuous state of inflammation. This cascade worsens the neural plaque load and accelerates the progression of the disease (Dong et al. 2009). To overcome this, a disease-modifying drug should also target neuro-inflammation (Coman and Nemeş, 2017). In the present study, SCO + HMM rats exhibited impaired memory along with significantly elevated hippocampal levels of pro-inflammatory cytokines. These findings strengthened the notion of the involvement of neuro-inflammation in the cognitive dysfunction and suggest its role in the dysregulated or increased levels AChE activity and subsequent behavioral alterations observed in demented rats. On the contrary, hippocampal levels of TNF-α and IL-1β were notably reduced in SCO + HMM rats treated with CUR and GBE combination. Again, the combination resulted in a more profound reduction in hippocampal TNF-α and IL-1β levels than CUR alone. Also, the study of Abdelmeguid et al (2021) revealed that the combined treatment of docosahexaenoic acid and GBE significantly decreased TNF-α level compared to diseased rats. All these findings suggest that GBE can improve the antioxidant and anti-inflammatory activity of CUR, and this could be attributed to enhancement of CUR brain levels by the co-administered GBE as confirmed by HPLC results.

Both experimental animals and AD patients' neurons displayed apoptotic alterations, such as caspase-3 activation (Forloni et al. 1993; Gastard et al. 2003; Kovacs et al. 1999; Płóciennik et al. 2015). This protease is thought to be crucial for apoptosis because it performs an irreversible phase in the apoptotic process (Wolf et al. 1999). In the current study, SCO + HMM-treated rats showed a significant increase in hippocampal caspase-3 levels. According to these findings, therapy with CUR and GBE combination dramatically prevented the elevation of hippocampal levels of caspase-3 compared to the SCO + HMM group. The combined effects of CUR and GBE were more pronounced than CUR alone. Similarly, Tian et al. (2013) demonstrated that hyperbaric oxygen and GBE reduced caspase-3 activity in the hippocampus of rats with AD induced by Aβ25–35 injection, and their combination showed a more significant effect than the individual treatments.

Another well-known fact is that AChE expression and activity control the dynamic concentration of ACh in the cholinergic synapses of the brain (Buckingham et al. 2009). The altered levels of AChE in AD patient’s brains and plasma, as well as the co-localization of this enzyme with Aβ deposits in the hippocampus, suggest that this enzyme may have a crucial role in the pathogenesis of AD (Mushtaq et al. 2014; de Matos et al. 2018). In the present study, AChE activity was remarkably increased in the hippocampal homogenates of SCO/HMM-treated rats, compared to their normal surrogates. Our result revealed that co-administration of CUR and GBE produced a synergistic inhibitory effect on AChE hippocampal activity as compared to the diseased group. This inhibitory effect was also more pronounced than those animals treated with CUR alone. In agreement to this finding, Zhao et al. (2021) found that diseased rats treated with low/high-dose donepezil and GBE had significantly lower AChE activities than those treated with each of the individual drugs.

Again, these findings support the idea that co-administration of GBE can enhance the beneficial effects of CUR on AD-like alterations, including anti-cholinesterase activity and caspase-3 level. This may also be the result of increasing central CUR levels.

Collectively, results of the current study suggest that a combination of CUR and GBE could ameliorate the learning and memory impairment in SCO + HMM diseased rats through the reversal of the AD-related neural and biochemical alterations. This combination dramatically enhanced the beneficial effects of CUR. The ability of the combination of CUR and GBE to delay the development or arrest the progression of SCO + HMM-induced AD requires further studies.

## Conclusion

Our results provide strong evidence for the hypothesis that ginkgo biloba extract increases curcumin's plasma bioavailability and brain access, which may improve curcumin’s effectiveness against characteristic symptoms of AD. Our study demonstrated that the combination of curcumin and Ginkgo biloba extract is superior to curcumin alone in preventing cognitive dysfunction, hippocampal neuronal degeneration and accumulation of intracellular NFT, and extracellular Aβ plaques in SCO + HMM-demented rats. In addition, the increased ability of curcumin by ginkgo biloba extract to attenuate cholinesterase activity, and enhance its anti-inflammatory and antioxidant properties, was evidenced. These effects may be mediated by activate gap junctions, improve the para-cellular permeability of the BBB via adenosine receptor activation and P-glycoprotein inhibitor of ginkgo biloba extract. This research may serve as a stepping stone to the creation of an innovative anti-neurodegeneration prophylactic strategy. Moreover, it may support the idea that ginkgo biloba extract and curcumin may be used together to prevent the central pathological changes that result in the occurrence of AD, and arrest the progression of AD successfully. Therefore, it is recommended that further studies should be applied to reveal the effectiveness of this combination strategy in treatment of the AD.

## Data Availability

The data that support the findings of this study are available from the corresponding author upon reasonable request. Some data may not be made available because of privacy or ethical restrictions.

## Abbreviations

AChE: Acetylcholinesterase
AD: Alzheimer’s disease
ANOVA: Analysis of variance
APP: Amyloid precursor protein
As: Arsenic
Aβ: Amyloid beta
BBB: Blood–brain barrier
Cd: Cadmium
CMC: Carboxymethyl cellulose
CUR: Curcumin
DI: Discrimination index
ELISA: Enzyme-linked immunosorbent assays
GBE: Ginkgo biloba extract
GSH: Reduced glutathione
HMM: Heavy metal mixture
HPF: High-power field
I.P.: Intraperitoneal
IHC: Immunohistochemistry
IL-1β: Interleukin-1β
MDA: Malondialdehyde
MEM: Memantine
MWM: Morris water maze
NC: Negative control
NFTs: Neurofibrillary tangles
NMDA: N-methyl-D-aspartate
NORT: Novel object recognition test
PA: Passive avoidance
Pb: Lead
PBS: Phosphate-buffered saline
p-tau: Phosphorylated tau protein
ROS: Reactive oxygen species
RP-HPLC: Reversed phase high-performance liquid column
SCO: Scopolamine
SE: Standard error
STL: Step-through latency
TNF-α: Tumor necrosis factor-α

## Declarations

### Author agreement

We the undersigned declare that this manuscript is original, has not been published before and is not currently being considered for publication elsewhere. We confirm that the manuscript has been read and approved by all named authors and that there are no other persons who satisfied the criteria for authorship but are not listed. We further confirm that the order of authors listed in the manuscript has been approved by all of us. We understand that the corresponding author is the sole contact for the Editorial process. He/she is responsible for communicating with the other authors about progress, submissions of revisions, and final approval of proofs.
